# Diagnostic Accuracy of Bone Turnover Markers in Patients With CKD: A Systematic Review and Meta-Analysis

**DOI:** 10.1016/j.xkme.2026.101309

**Published:** 2026-02-25

**Authors:** Jeerath Phannajit, Kavita Jintanapramote, Kullaya Takkavatakarn, Aschariya Wipattanakitcharoen, Kearkiat Praditpornsilpa, Somchai Eiam-Ong, Paweena Susantitaphong

**Affiliations:** 1 Center of Excellence for Metabolic Bone Disease in CKD patients, Faculty of Medicine, Chulalongkorn University, Bangkok, Thailand; 2 Division of Clinical Epidemiology, Department of Medicine, King Chulalongkorn Memorial Hospital, Thai Red Cross Society, Bangkok, Thailand; 3 Division of Nephrology, Department of Medicine, Faculty of Medicine, Chulalongkorn University, Bangkok, Thailand; 4 Division of Nephrology, Department of Medicine, Bhumibol Aduldej Hospital, The Royal Thai Air Force, Bangkok, Thailand

**Keywords:** chronic kidney disease (CKD), renal failure, renal osteodystrophy (ROD), CKD–mineral and bone disorder (CKD–MBD), bone turnover markers (BTMs), biochemical markers of bone metabolism, bone histomorphometry, bone biopsy, diagnostic accuracy, receiver operating characteristic (ROC) curve

## Abstract

**Rationale & Objective:**

Abnormal bone turnover in chronic kidney disease (CKD) increases fracture risk and influences treatment response, making accurate assessment essential. Although bone histomorphometry is the diagnostic gold standard, its invasiveness limits routine use. This study evaluated the diagnostic accuracy of noninvasive bone turnover markers—tartrate-resistant acid phosphatase 5b (TRAP5b), bone-specific alkaline phosphatase (BALP), and procollagen type I N-terminal propeptide (PINP)—compared with parathyroid hormone (PTH) for detecting high or low bone turnover in CKD.

**Study Design:**

A systematic review and meta-analysis of observational studies.

**Setting & Study Populations:**

Adult patients with CKD (all stages).

**Selection Criteria for Studies:**

Studies were included if they compared serum bone turnover markers to bone histomorphometry.

**Data Extraction:**

Two reviewers independently extracted study characteristics, population details, assay types, cutoff thresholds, sensitivity, specificity, and area under the ROC curve (AUC).

**Analytical Approach:**

A meta-analysis using random-effects models pooled AUCs for detecting high and low bone turnover per marker including intact PTH. Subgroup analyses assessed assay types (mass-based vs activity-based BALP, intact vs total PINP) and CKD stages.

**Results:**

Eight studies were included with 6 studies providing AUC data for pooling. Intact PINP and BALP (mass assay) demonstrated the highest accuracy for identifying high bone turnover, whereas intact PTH showed only moderate discriminatory performance. For low turnover, TRAP5b, BALP, and intact PINP displayed comparable accuracy, while total PINP and PTH were less reliable. Reported diagnostic thresholds varied but generally yielded sensitivities of 50%-90% and specificities of 60%-95%. One study suggested that combining bone turnover markers, such as PINP with BALP or TRAP5b, may further enhance diagnostic discrimination.

**Limitations:**

Limited representation of non-hemodialysis populations, small sample sizes, and lack of standardized methods to determine thresholds

**Conclusions:**

TRAP5b, BALP, and intact PINP demonstrated moderate diagnostic accuracy and may support treatment decisions in CKD when bone biopsy is not feasible. (PROSPERO: CRD420251003120)

## Plain-Language Summary

People with chronic kidney disease often have weak or brittle bones because their bone turnover—the balance between bone breakdown and formation—can be too high or too low. The usual way to check this is with a bone biopsy, but that test is invasive and rarely done. Our study reviewed research on 3 blood tests, called tartrate-resistant acid phosphatase 5b, bone-specific alkaline phosphatase, and procollagen type I N-terminal propeptide, to see if they can show bone turnover more accurately than the common parathyroid hormone test. By combining results from several studies, we found that these newer blood markers were more reliable and could better identify bone problems. These findings suggest that simple blood tests may help guide safer and more effective treatment for bone disease in kidney patients.

Fractures are a major complication of chronic kidney disease–mineral and bone disorder (CKD-MBD), historically termed renal osteodystrophy.^1^ There is currently increasing recognition of CKD-associated osteoporosis as a distinct clinical entity, particularly among patients with CKD stages 4-5 and those on dialysis, where fracture risk is markedly elevated.^1^, ^2^, ^3^, ^4^, ^5^ This skeletal fragility results from abnormalities in bone turnover, mineralization, and structure, contributing to increased morbidity, cardiovascular events, and premature death.

Accurate assessment of bone turnover is essential, as treatment decisions depend heavily on the underlying turnover state, including the management of CKD-MBD and secondary hyperparathyroidism through optimization or adjustment of active vitamin D and calcimimetic therapy, maintenance of calcium and phosphate balance, and the selection of antiresorptive or anabolic agents for osteoporosis. Bone biopsy with tetracycline-labeled trans-iliac histomorphometry remains the gold standard for evaluating bone turnover and guiding therapy.^2^^,^^6^, ^7^, ^8^ However, this invasive procedure is limited by availability, technical demands, and patient acceptability. In this context, non-invasive biomarkers of bone turnover have gained clinical interest.

Bone turnover markers (BTMs) offer a promising alternative due to their accessibility and noninvasiveness. Bone-specific alkaline phosphatase (BALP), procollagen type I N-terminal propeptide (PINP), and tartrate-resistant acid phosphatase 5b (TRAP5b) are particularly noteworthy for their low renal clearance, making them suitable candidates in population with CKD.^9^^,^^10^ In contrast, traditional reliance on intact parathyroid hormone (intact PTH) is limited by poor specificity and biological variability across patients and assay types.^11^

Although several studies have assessed the utility of BTMs in predicting bone turnover states among patients with CKD, the findings are heterogeneous, varying by marker, assay methodology, and patient population. Recent international consensus papers, including the 2024 KDIGO Controversies Conference on CKD–MBD and the 2025 multi-stakeholder consensus, have emphasized the clinical importance of bone turnover assessment and recommended non-renally cleared BTMs such as BALP, PINP, and TRAP5b. However, these recommendations are based largely on expert opinion rather than quantitative diagnostic validation. This systematic review and meta-analysis address that gap by evaluating the diagnostic performance of BALP, PINP, and TRAP5b for identifying high and low bone turnover in CKD using bone histomorphometry as the reference standard, aiming to provide pooled estimates that can guide clinical decision-making and support non-invasive diagnostic strategies for bone disease in CKD.

## Methods

### Search Strategy

A comprehensive electronic search was conducted across PubMed, Scopus, EMBASE, ClinicalTrials.gov, and the World Health Organization International Clinical Trials Registry Platform (WHO-ICTRP) to identify relevant studies published in English from database inception through March 3, 2025. The search strategy was collaboratively developed and tailored to each database, with the complete PubMed terms provided in Item S1. Additional sources were identified by reviewing reference lists of included articles and consulting field experts. The study protocol was prospectively registered in PROSPERO (CRD420251003120). This review followed PRISMA 2020 guidelines for reporting systematic reviews.^12^ The Meta-analyses of Observational Studies in Epidemiology (MOOSE) checklist^13^ is provided in Item S2.

### Study Screening and Selection

Eligible studies were cross-sectional, case-control, or cohort designs that reported at least one diagnostic accuracy parameter, such as sensitivity, specificity, or area under the receiver operating characteristic curve (AUC). The index tests of interest included BALP, PINP, and TRAP5b, which represent direct biochemical markers of bone formation or resorption. Intact parathyroid hormone (intact PTH, 2nd generation assay) was additionally included as a comparator, given its indirect reflection of bone turnover through regulation of bone remodeling and its status as the conventional and most commonly used marker in routine clinical practice. The reference standard for determining bone turnover status was bone histomorphometry, obtained from trans-iliac bone biopsy following a double course of tetracycline labeling. Two authors (J.P. and K.J.) independently screened titles and abstracts, followed by full-text review. Discrepancies were resolved through discussion with a third author (P.S.).

### Data Extraction and Methodological Quality Assessment

All references were managed using EndNote X9, and study screening was conducted via Covidence. Data extraction was performed using Microsoft Excel 365. From each included study, data were extracted to construct 2×2 contingency tables (true positives, false positives, true negatives, and false negatives), along with reported area under the ROC curve (AUC) values and their 95% confidence intervals (CIs). Additional information such as year of publication, bone turnover prevalence, and histomorphometric diagnostic categories was also collected. Data extraction was conducted independently by three authors (J.P., K.J., and A.W.).

Methodological quality and risk of bias were assessed using the Quality Assessment of Diagnostic Accuracy Studies, version 2 (QUADAS-2) tool which evaluates 4 domains: patient selection, index test, reference standard, and flow and timing. Two authors (J.P. and K.J.) independently performed the quality assessment, with disagreements resolved by consensus. If standard errors were not directly reported, they were calculated from confidence intervals or derived using methods recommended by the Cochrane Handbook for Systematic Reviews of Diagnostic Test Accuracy.^14^

### Data Synthesis and Analysis

Quantitative synthesis was performed in Stata SE 18.0. The primary analysis pooled area under the receiver operating characteristic (ROC) curve (AUC) using random-effects meta-analysis, including only studies reporting AUC with corresponding standard errors. Extracted AUCs and their 95% CIs were transformed to the logit scale to stabilize variances, and corresponding standard errors were calculated from the CI limits. Random-effects meta-analysis was performed using the restricted maximum likelihood method to pool logit-transformed AUCs, followed by back-transformation to the original AUC scale for interpretation. Analyses were stratified by bone turnover marker, assay types, and diagnostic category (high vs nonhigh and low vs non-low bone turnover). Between-study heterogeneity was assessed using I^2^ statistic. Reporting bias was not formally assessed due to the limited number of included studies per marker. This study was based exclusively on previously published data and did not involve the collection or use of individual patient information. As such, it was exempted from formal approval by an institutional review board, and informed consent was not applicable due to no need to contact individuals or access identifiable data.

## Results

### Study Characteristics and Quality Assessment

**Figure 1:**
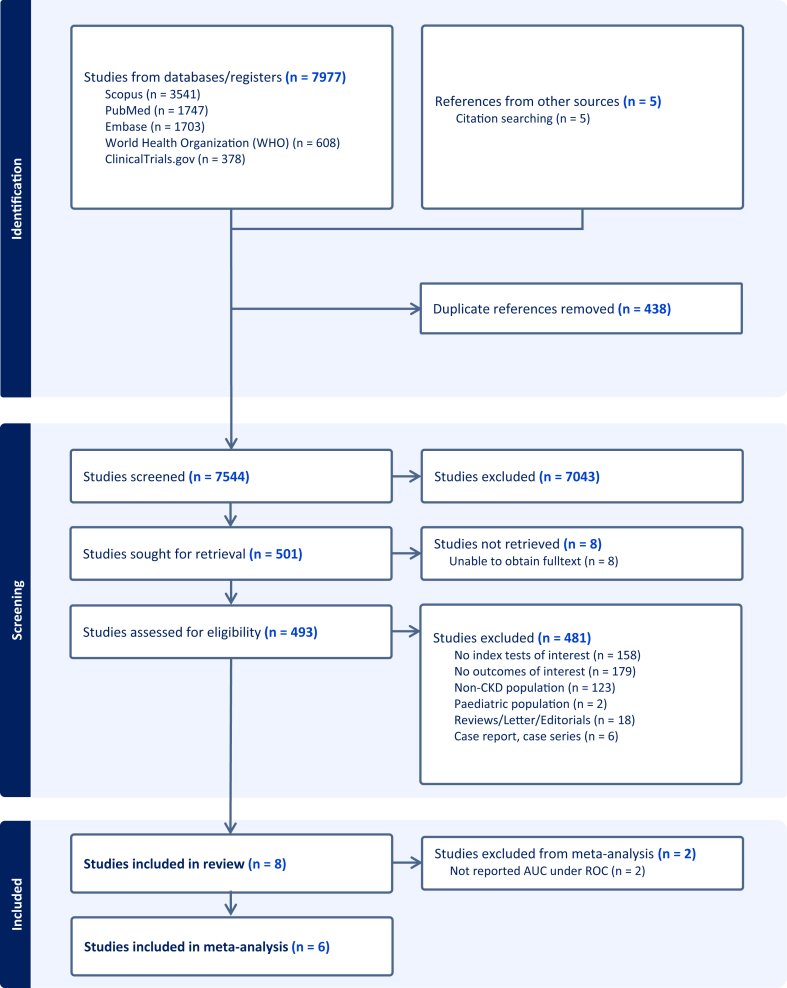
PRISMA Diagram.

Following a comprehensive database search and de-duplication, a total of 7,544 titles and abstracts were screened for eligibility, leading to the retrieval of 501 full-text articles for full-text review. Of these, eight studies^11^^,^^15^, ^16^, ^17^, ^18^, ^19^, ^20^, ^21^ met the inclusion criteria for the systematic review. Among them, 6 studies^11^^,^^16^^,^^18^, ^19^, ^20^, ^21^ reported the AUC and were included in the meta-analysis. The selection process is illustrated in Fig 1, in accordance with the PRISMA guidelines.

The majority of included studies enrolled patients with advanced chronic kidney disease (stage G3–G5D), with 6^11^^,^^15^^,^^17^^,^^19^^,^^21^ studies comprised of exclusively or predominantly hemodialysis populations. Four studies^16^, ^17^, ^18^, ^19^, ^20^, ^21^ included pre-dialysis patients with CKD and one study^16^ included kidney transplant patients. Most studies described their populations as mixed, without stratifying results by treatment modality. The reported prevalence of bone turnover categories varied across studies. The proportion of patients with low bone turnover ranged from 11.5% to 58.7%, and high bone turnover from 16.9% to 81.3%. A distinct normal bone turnover subgroup was reported in 5 studies,^16^^,^^18^, ^19^, ^20^, ^21^ with proportions ranging from 12.6% to 54.8% (Table 1).

**Table 1:** Characteristics of Studies and Prevalence of Bone Turnover According to Bone Histomorphometry

Reference	Country/Region	Population	N	Histomorphometry Diagnosis			Reported Markers			
				Low Turnover (%)	Normal Turnover(%)	High Turnover (%)	BALP	PINP	TRAP5b	iPTH
Coen et al^15^ (1998)	Italy	CKD G5D (all HD)	41	22.0%	-	78.0%	✓(mass)	-	-	✓^b^
Lehmann et al^17^ (2008)^a^	Germany	CKD stage G3-5ND (n = 36) CKD G5D (n = 96, 95% HD)	132	12.1%	-	77.3%	✓(mass)	-	✓	✓^b^
Sprague et al^20^ (2016)	Brazil, Portugal, Turkey, and Venezuela	CKD G5D (93% HD, 7% PD)	492	58.7%	24.4%	16.9%	✓(activity)	✓ (Total)	-	✓
Salam et al^19^ (2018)	UK	CKD stage G4-5ND (n = 44) CKD G5D (n = 25)	69	15.9%	21.7%	24.6%	✓(mass)	✓(intact + total)	✓	✓
Lima et al^18^ (2019)^a^	USA	CKD stage G2-5ND (70) CKD G5D (n = 34, all HD)	104	54.8%	12.5%	32.7%	✓(activity)	-	✓	✓
Laowalert et al^11^ (2020)	Thailand	CKD G5D (all HD)	22	45.5%	-	54.5%	✓(activity)	-	✓	✓
Ursem et al^21^ (2021)	Belgium	CKD G5D (58% HD)	31	32%	32%	35%	✓	✓	✓	✓
Jørgensen et al^16^ (2022)^a^	Europe	CKD G4-5ND (14) CKD G5D (n = 66, 67% HD) KT (n = 199)	199	16.6%	54.8%	28.6%	✓(mass)	✓(intact)	✓	(biointact 1-84)

Abbreviations: BALP, bone specific alkaline phosphatase; D, dialysis; HD, hemodialysis; iPTH, intact parathyroid hormone; KT, kidney transplantation; ND, non-dialysis; PD, peritoneal dialysis; PINP, procollagen type I N terminal propeptide; TRAP-5b, tartrate resistant acid phosphatase type 5b.

a Reported mixed results among predialysis and patients treated with dialysis.

b Not included in meta-analysis because of no AUC or its standard error reported.

**Figure 2:**
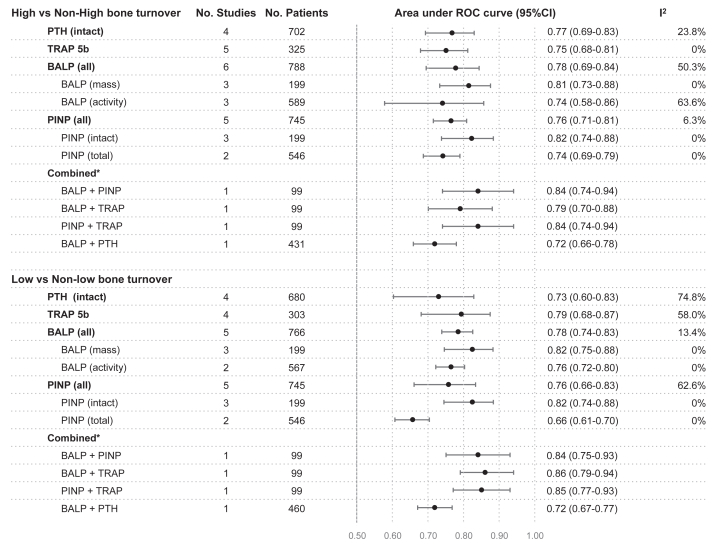
Performance of bone turnover markers in detecting high and low bone turnover status. BALP, bone specific alkaline phosphatase; PTH, parathyroid hormone; PINP, procollagen type I N terminal propeptide; ROC curve, receiver operating characteristic curve; TRAP-5b, tartrate resistant acid phosphatase type 5b. ^a^Each combination of combined bone turnover markers results was extracted from single study.

The assay techniques used for bone turnover marker measurements varied across studies (Table S1). The methodological quality of the studies was assessed using the QUADAS-2 tool (Fig 2). All seven studies were rated as having a high risk of bias in the index test and reference standard domain. The patient selection domain was rated as low risk in 4 studies^11^^,^^16^^,^^17^^,^^20^ and unclear in 3. The flow and timing domain was rated as low risk in 6 studies^11^^,^^16^, ^17^, ^18^, ^19^, ^20^ and unclear in one.^15^ (Table S2).

### Performance of Bone turnover makers and intact PTH in Detecting High and Low Bone Turnover Status

Among the 6 studies included in the meta-analysis, the diagnostic performance of the bone turnover markers TRAP5b, BALP, and PINP, along with intact PTH as a comparator, was evaluated for differentiating both high versus nonhigh and low versus nonlow bone turnover (Fig 2 and Figs S1-S8). One study additionally assessed the performance of combined marker panels.

For distinguishing high versus nonhigh bone turnover, intact PINP and BALP (mass assay) demonstrated the highest diagnostic accuracy among all markers, whereas intact PTH showed only moderate discrimination. One study further reported that combining PINP with BALP or TRAP5b provided additional discriminatory power (Fig 2).

For identifying low versus nonlow bone turnover, TRAP5b, BALP (mass assay), and intact PINP exhibited comparable performance, while intact PTH and total PINP showed limited ability to discriminate between groups. Combinations of BALP, PINP, and TRAP5b were associated with further improvement in diagnostic accuracy (Fig 2).

### Reported Diagnostic Thresholds of Bone Turnover Markers and PTH

Cutoff values used to differentiate high and low bone turnover varied substantially across studies (Tables S3-S4). Reported thresholds for intact PTH ranged from ∼80 to 630 pg/mL for identifying high turnover, with sensitivity ranging from 50% to 92% and specificity from 50% to 100%, while thresholds below 80-100 pg/mL were associated with low turnover (sensitivity 89%, specificity 94%, reported in one study). For TRAP5b, optimal thresholds generally ranged from 1.2 to 5.0 U/L for high turnover (sensitivity 52%-83%, specificity 58%-100%) and below 3-–4 U/L for low turnover (sensitivity 56%-89%, specificity 71%-80%). BALP thresholds varied between 23-46 U/L for activity assays and 33-42 μg/L for mass assays, showing sensitivity of 10%-72% and 56%-100% and specificity of 56%-84% and 81%-94%, respectively. Reported PINP thresholds were approximately 50-120 ng/mL for intact assays and 120-500 ng/mL for total assays, corresponding to sensitivity of 53%-94% and specificity of 68%-94% for high turnover, while values below 50-60 ng/mL were indicative of low turnover (sensitivity 80%, specificity 70%-75%).

## Discussion

This systematic review and meta-analysis evaluated the diagnostic accuracy of novel BTMs for assessing bone turnover status in CKD. The 3 non-renally cleared markers—TRAP5b, BALP, and PINP—showed moderate to good diagnostic performance, outperforming PTH in discriminating turnover states. Intact PINP and BALP (mass assays) demonstrated slightly higher accuracy than total or activity-based assays, underscoring the clinical importance of assay type.

Accurate assessment of bone turnover is essential for guiding therapy and preventing inappropriate treatment in CKD–MBD.^1^^,^^9^^,^^10^ Although PTH remains the most accessible biomarker, our analysis confirmed its limited diagnostic accuracy compared with bone histomorphometry and non-renally cleared BTMs. These findings are consistent with international consensus statements (e.g. KDIGO and ESECO/IOF/IFCC),^1^^,^^9^ which emphasize that PTH is a regulator of bone metabolism rather than a direct turnover marker and should be interpreted in conjunction with calcium, phosphate, vitamin D, and BTMs.

The superior performance of intact PINP, BALP, and TRAP5b likely reflects their non-renal clearance, allowing consistent interpretation across different kidney-function levels. One study^16^ suggests additive value when BTMs are combined (eg, PINP + TRAP5b or BALP), though this remains to be confirmed in larger studies.

Because of heterogeneity in threshold definitions and the limited number of studies, formal meta-analysis of diagnostic cut-offs was not feasible. Based on the best available evidence and for clinical simplicity, approximate reference ranges suggest BALP < 20 or > 35 μg/L, intact PINP < 50 or > 120 μg/L, and TRAP5b < 3.5 or > 5.0 U/L to indicate low and high bone turnover, respectively. Intact PTH (2nd-generation assay) < 180 or > 327 pg/mL may also be considered, though interpretation should be made with caution due to its lower diagnostic accuracy. These ranges are consistent with the 2024 KDIGO Controversies Conference recommendations and may help guide clinical interpretation when a bone biopsy is not feasible.^1^

This review provides focused quantitative synthesis to date of BTMs validated against bone histomorphometry in CKD. By including only studies reporting AUCs and stratifying by assay type and turnover category, the analysis offers a robust, standardized summary of diagnostic accuracy. Methodological quality was assessed using QUADAS-2, which identified consistent risks of bias in blinding and threshold prespecification.

Limitations include the small number of studies per marker, predominance of hemodialysis cohorts, and underrepresentation of peritoneal dialysis and transplant populations. Incomplete reporting of diagnostic thresholds also reduced precision. Further studies are warranted to validate these findings across CKD populations, establish standardized cut-offs, and assess the clinical and cost-effectiveness of BTM-guided management.

In conclusion, novel bone turnover markers (TRAP5b, BALP, and intact PINP) showed promising diagnostic accuracy for assessing bone turnover in CKD, especially when measured using non-renally cleared assays. These markers represent practical, minimally invasive alternatives to bone biopsy. Future research should aim to standardize assays, refine diagnostic thresholds, and validate their clinical utility across dialysis modalities and transplant settings.

## Supplementary Materials

Supplementary File (PDF) Figure S1-S8; Items S1-S2; Table S1-S4.

## Footnotes

Supplementary File (PDF)
**Figure S1:** Meta-analysis of TRAP5b for differentiating high vs non-high bone turnover status. **Figure S2:** Meta-analysis of TRAP5b for differentiating low vs non- low bone turnover status. **Figure S3:** Meta-analysis of BALP for differentiating high vs non-high bone turnover status. **Figure S4:** Meta-analysis of BALP for differentiating low vs non- low bone turnover status. **Figure S5:** Meta-analysis of PINP for differentiating high vs non-high bone turnover status. **Figure S6:** Meta-analysis of SPINP for differentiating low vs non- low bone turnover status. **Figure S7:** Meta-analysis of intact PTH for differentiating high vs non-high bone turnover status. **Figure S8:** Meta-analysis of intact PTH for differentiating low vs non- low bone turnover status. **Item S1:** Search terms. **Item S2:** Meta-analyses Of Observational Studies in Epidemiology (MOOSE) checklist. **Table S1:** Laboratory Techniques, Assays, and Reference Ranges of BTMs Reported in Each Study. **Table S2:** Methodological Quality Assessments Based on the QUODAS-2 Domains. **Table S3:** Sensitivity and Specificity of BTMs in Suggested Cutoff(s) to Differentiate High and non-High Bone Turnover Reported in Each Study. **Table S4:** Sensitivity and Specificity of BTMs in Suggested Cutoff(s) to Differentiate low and non-low Bone Turnover Reported in Each Study.
