# Supplementary material for: Diagnostic Accuracy of Bone Turnover Markers in Patients With CKD: A Systematic Review and Meta-Analysis
Source: Kidney Med. 2026 Feb 25;8(5):101309. doi: 10.1016/j.xkme.2026.101309 (PMC13087464; doi:10.1016/j.xkme.2026.101309)
Supplement: Supplementary File (PDF) — Figure S1-S8; Items S1-S2; Table S1-S4. [file mmc1.pdf]

**Item S1: Search terms**

PubMed:

| |
|---|
| (BALP OR PINP OR sclerotic OR TRAP5B OR PTH OR BONE TURNOVER OR BONE HISTOMORPHOMETRY OR BONE BIOPSY OR osteoporosis OR FRACTURE OR BONE DENSITY) AND (CKD OR ESRD OR DIALYSIS) Filters: Clinical Study, Clinical Trial, Clinical Trial, Phase I, Clinical Trial, Phase II, Clinical Trial, Phase III, Clinical Trial, Phase IV, Comparative Study, Controlled Clinical Trial, Multicenter Study, Observational Study, Randomized Controlled Trial |

Scopus:

| |
|---|
| TITLE-ABS-KEY(("BALP" OR "PINP" OR "TRAP5B" OR "PTH" OR "bone turnover" OR "bone remodeling" OR ("bone" AND "histomorphometry") OR ("bone" AND ("biopsy" OR "biopsies" OR "pathology")) OR "osteoporosis" OR "fracture" OR "bone density")<br>AND<br>("CKD" OR "chronic kidney disease" OR "chronic kidney failure" OR "ESRD" OR "dialysis" OR "renal dialysis")) |

EMBASE:

| |
|---|
| ('bone turnover'/exp OR 'bone turnover' OR 'bone remodeling'/exp OR 'bone remodeling' OR 'BALP' OR 'PINP' OR 'TRAP5b' OR 'PTH' OR 'bone biopsy'/exp OR 'bone biopsy' OR 'bone histomorphometry' OR 'osteoporosis'/exp OR 'osteoporosis' OR 'fracture'/exp OR 'fracture' OR 'bone density'/exp OR 'bone density')<br>AND<br>('chronic kidney disease'/exp OR 'CKD' OR 'chronic kidney failure' OR 'ESRD' OR 'dialysis'/exp OR 'dialysis' OR 'renal dialysis') |

ClinicalTrials.gov

| |
|---|
| Condition or Disease: Chronic Kidney Disease OR Dialysis<br>Other Terms: BALP OR PINP OR TRAP5B OR PTH OR "bone turnover" OR "bone remodeling" OR histomorphometry OR biopsy OR osteoporosis OR fracture OR "bone density" |

WHO ICTRP

| |
|---|
| BALP OR PINP OR TRAP5B OR PTH OR "bone turnover" OR "bone remodeling" OR histomorphometry OR biopsy OR osteoporosis OR fracture OR "bone density"<br>AND Chronic Kidney Disease OR CKD OR ESRD OR dialysis OR "renal dialysis" |

**Item S2: Meta-analyses Of Observational Studies in Epidemiology (MOOSE) Checklist**

| Item No | Recommendation | Reported on Page No |
|---|---|---|
| Reporting of background should include | | |
| 1 | Problem definition | 4-5 |
| 2 | Hypothesis statement | 5 |
| 3 | Description of study outcome(s) | 5 |
| 4 | Type of exposure or intervention used | 5-6 |
| 5 | Type of study designs used | 5-6 |
| 6 | Study population | 5 |
| Reporting of search strategy should include | | |
| 7 | Qualifications of searchers (eg, librarians and investigators) | 5-6 |
| 8 | Search strategy, including time period included in the synthesis and key words | 5 |
| 9 | Effort to include all available studies, including contact with authors | 5 |
| 10 | Databases and registries searched | 5 |
| 11 | Search software used, name and version, including special features used (eg, explosion) | 5 |
| 12 | Use of hand searching (eg, reference lists of obtained articles) | 5 |
| 13 | List of citations located and those excluded, including justification | Fig 1 |
| 14 | Method of addressing articles published in languages other than English | 5-6 |
| 15 | Method of handling abstracts and unpublished studies | 5 |
| 16 | Description of any contact with authors | N/A |
| Reporting of methods should include | | |
| 17 | Description of relevance or appropriateness of studies assembled for assessing the hypothesis to be tested | 6-7 |
| 18 | Rationale for the selection and coding of data (eg, sound clinical principles or convenience) | 6-7 |
| 19 | Documentation of how data were classified and coded (eg, multiple raters, blinding and interrater reliability) | N/A |
| 20 | Assessment of confounding (eg, comparability of cases and controls in studies where appropriate) | 6-7 |
| 21 | Assessment of study quality, including blinding of quality assessors, stratification or regression on possible predictors of study results | 6-7 |
| 22 | Assessment of heterogeneity | 6-7 |
| 23 | Description of statistical methods (eg, complete description of fixed or random effects models, justification of whether the chosen models account for predictors of study results, dose-response models, or cumulative meta-analysis) in sufficient detail to be replicated | 6-7 |
| 24 | Provision of appropriate tables and graphics | Tables 1, Figs 1, S1-S8 |
| Reporting of results should include | | |
| 25 | Graphic summarizing individual study estimates and overall estimate | Figs S1-S8 |
| 26 | Table giving descriptive information for each study included | Table 1 |
| 27 | Results of sensitivity testing (eg, subgroup analysis) | Figs 1, S1-S8 |
| 28 | Indication of statistical uncertainty of findings | Figs 1, S1-S8 |
| Reporting of discussion should include | | |
| 29 | Quantitative assessment of bias (eg, publication bias) | N/A |
| 30 | Justification for exclusion (eg, exclusion of non-English language citations) | 10-11 |
| 31 | Assessment of quality of included studies | 10-11 |
| Reporting of conclusions should include | | |
| 32 | Consideration of alternative explanations for observed results | 10-11 |
| 33 | Generalization of the conclusions (ie, appropriate for the data presented and within the domain of the literature review) | 10-11 |
| 34 | Guidelines for future research | 10-11 |
| 35 | Disclosure of funding source | 13 |

From: Stroup DF, Berlin JA, Morton SC, et al, for the Meta-analysis Of Observational Studies in Epidemiology (MOOSE) Group. Meta-analysis of Observational Studies in Epidemiology. A Proposal for Reporting. JAMA. 2000;283(15):2008-2012. doi: 10.1001/jama.283.15.2008.

**Table S1** Laboratory techniques, assays, and reference ranges of BTMs reported in each study

| | **BALP** | **PINP** | **TRAP5b** | **Intact PTH** |
|---|---|---|---|---|
| **Coen (1998)** | Mass, EIA<br>Hybritech (11.8±4.3 ng/mL) | *Not reported* | *Not reported* | IRMA (2nd generation)<br>(double antibody,<br>Incstar, USA, 15-55 pg/mL) |
| **Laowalert (2020)** | Activity, EIA<br>Quidel (15–41.3 U/L) | *Not reported* | EIA<br>Quidel (unspecified U/L) | CLIA (2nd generation)<br>(Roche, 15-65 pg/mL) |
| **Sprague (2016)** | Activity, EIA<br>Quidel (15–41.3 U/L) | Total, EIA<br>Not specified (13.9–85.5 ng/mL) | *Not reported* | CLIA (2nd generation)<br>(Roche, 15-65 pg/mL) |
| **Lehmann (2008)** | Mass, not specified<br>(8.5–17.9µg/l) | *Not reported* | EIA<br>Wedel (3.4±1.3U/l) | CLIA (2nd generation) (Nichols, USA, 10-65 pg/mL) |
| **Salam (2018)** | Mass, CLIA<br>IDS-iSYS (6.1–25.5 µg/L) | Intact (trimeric), CLIA<br>IDS-iSYS (12.8–82.6 ng/mL)<br><br>Total, EIA<br>Roche (unspecified ng/mL) | CLIA<br>IDS-iSYS (1.1-6.9 U/L) | CLIA (2nd generation)<br>(IDS, unspecified range) |
| **Lima (2019)** | Activity, EIA<br>Quidel (15–41.3 U/L) | *Not reported* | EIA<br>Quidel (unspecified U/L) | CLIA (2nd generation)<br>(Diasorin, USA, , unspecified range) |
| **Ursem (2021)** | Mass, CLIA<br>IDS-iSYS (6.1–25.5 µg/L) | Intact (trimeric), CLIA<br>IDS-iSYS (12.8–82.6 ng/mL) | CLIA<br>IDS-iSYS (1.1-6.9 U/L) | CLIA (2nd generation)<br>(Roche, 15-65 pg/mL) |
| **Jorgensen (2022)** | Mass, CLIA<br>IDS-iSYS (6.1–25.5 µg/L) | Intact (trimeric), CLIA<br>IDS-iSYS (12.8–82.6 ng/mL) | CLIA<br>IDS-iSYS (1.1-6.9 U/L) | *biointact (1-84) PTH, using in-house IRMA* |

Abbreviations: BALP; Bone specific alkaline phosphatase, TRAP-5b; Tartrate resistant acid phosphatase type 5b, PINP; procollagen type I N terminal propeptide, iPTH; intact parathyroid hormone, EIA; Enzyme immunoassay, CLIA; Chemiluminessence immunoassay, IRMA; Immunoradiometric assay.

**Table S2** Methodological quality assessments based on the QUODAS-2 domains

| QUODAS-2: Domains | Coen (1998) | Lehmann (2008) | Sprague (2016) | Salam (2018) | Lima (2019) | Laowalert (2020) | Ursem (2021) | Jorgensen (2022) |
|---|---|---|---|---|---|---|---|---|
| **PATIENT SELECTION** | | | | | | | | |
| Was a consecutive or random sample of patients enrolled? | U | Y | Y | Y | N | N | N | Y |
| Was a case-control design avoided? | Y | Y | Y | Y | Y | Y | N | Y |
| Were inappropriate exclusions avoided? | U | U | U | Y | Y | Y | Y | Y |
| **INDEX TEST** | | | | | | | | |
| Were the index test results interpreted without knowledge of the results of the reference standard? | U | Y | Y | Y | Y | U | U | U |
| If a threshold was used, was it pre-specified? | NA | NA | NA | NA | NA | NA | NA | NA |
| **REFERENCE STANDARD** | | | | | | | | |
| Is the reference standard likely to correctly classify the target condition? | Y | Y | Y | Y | Y | Y | N | Y |
| Were the reference standard results interpreted without knowledge of the results of the index test? | U | Y | Y | Y | U | U | U | U |
| **FLOW AND TIMING** | | | | | | | | |
| Was there an appropriate interval between index test(s) and reference standard? | Y | Y | Y | Y | Y | Y | Y | Y |
| Did all patients receive a reference standard? | Y | Y | Y | Y | Y | Y | Y | Y |
| Did all patients receive the same reference standard? | N | N | Y | Y | Y | Y | Y | Y |
| Were all patients included in the analysis? | Y | Y | Y | Y | Y | Y | Y | Y |
| **SUMMARY - RISK of BIAS** | | | | | | | | |
| PATIENT SELECTION | UNCLEAR | LOW | LOW | LOW | INTERMEDIATE | LOW | HIGH | LOW |
| INDEX TEST | UNCLEAR | LOW | LOW | LOW | LOW | HIGH | UNCLEAR | LOW |
| REFERENCE STANDARD | LOW | LOW | LOW | LOW | LOW | LOW | HIGH | LOW |
| FLOW AND TIMING | INTERMEDIATE | INTERMEDIATE | LOW | LOW | LOW | LOW | LOW | LOW |

Abbriviations: Y = Yes, N = No, U = Unclear, N/A = not applicable

**Table S3** Sensitivity and specificity of BTMs in suggested cutoff(s) to differentiate high and non-high bone turnover reported in each study

| Markers / study | Cutoff | Unit | Sensitivity | Specificity |
|---|---|---|---|---|
| **Intact PTH (*dialysis only)** | | | | |
| Coen (1998) | > 79.7 | pg/mL | 89% | 94% |
| Lehmann (2008)-CKD5D | > 161.5 | pg/mL | 75% | 78% |
| Sprague (2016) | > 323 | pg/mL | Not reported | Not reported |
| Laowalert (2020) | > 347 | pg/mL | 92% | 50% |
| Laowalert (2020) | > 484 | pg/mL | 75% | 80% |
| Laowalert (2020) | > 570 | pg/mL | 58% | 90% |
| Laowalert (2020) | > 633 | pg/mL | 50% | 100% |
| **Biointact (1-84) PTH** | | | | |
| Jorgensen (2022) | > 143.5 | pg/mL | 70% | 74% |
| **TRAP 5b** | | | | |
| Jorgensen (2022) | > 5.05 | U/L | 77% | 76% |
| Laowalert (2020) | > 1.91 | U/L | 75% | 70% |
| Laowalert (2020) | > 1.22 | U/L | 83% | 50% |
| Laowalert (2020) | > 2.67 | U/L | 58% | 90% |
| Lima (2019) | > 4.3 | U/L | 65% | 76% |
| Salam (2018) | > 4.6 | U/L | 81% | 58% |
| Lehmann (2008)-CKD5D | > 2.29 | U/L | 52% | 100% |
| **BALP** | | | | |
| Laowalert (2020) | > 26.94 | U/L (activity) | 60% | 67% |
| Laowalert (2020) | > 23.91 | U/L (activity) | 70% | 56% |
| Laowalert (2020) | > 46.14 | U/L (activity) | 10% | 78% |
| Lima (2019) | > 35 | U/L (activity) | 72% | 84% |
| Sprague (2016) | > 42.1 | U/L (activity) | Not reported | Not reported |
| Jorgensen (2022) | > 33.7 | ug/L (mass) | 73% | 86% |
| Salam (2018) | > 42.1 | ug/L (mass) | 56% | 83% |
| Lehmann (2008)-CKD5D | > 42.1 | ug/L (mass) | 71% | 81% |
| Coen (1998) | > 42.1 | ug/L (mass) | 100% | 94% |
| **PINP** | | | | |
| Jorgensen (2022) | > 120.7 | ng/mL (intact) | 73% | 94% |
| Salam (2018) | > 107 | ng/mL (intact) | 53% | 92% |
| Salam (2018) | > 142 | ng/mL (total) | 75% | 68% |
| Sprague (2016) | > 621.1 | ng/mL (total) | Not reported | Not reported |
| **Combined** | | | | |
| Sprague (2016) | combined BALP + intact PTH | | Not reported | Not reported |
| Jorgensen (2022) | combined BALP + PINP (intact) | | 63% | 97% |
| Jorgensen (2022) | combined BALP + TRAP | | 63% | 91% |
| Jorgensen (2022) | combined PINP (intact) + TRAP | | 82% | 94% |

**Table S4** Sensitivity and specificity of BTMs in suggested cutoff(s) to differentiate low and non-low bone turnover reported in each study

| Markers / study | Cutoff | Unit | Sensitivity | Specificity |
|---|---|---|---|---|
| **Intact PTH (*dialysis only)** | | | | |
| Coen (1998) | < 79.7 | pg/mL | 89% | 94% |
| Sprague (2016) | < 103.8 | pg/mL | Not reported | Not reported |
| **Biointact (1-84) PTH** | | | | |
| Jorgensen (2022) | <90.5 | pg/mL | 69% | 52% |
| **TRAP 5b** | | | | |
| Jorgensen (2022) | < 3.44 | U/L | 73% | 74% |
| Lima (2019) | < 4.3 | U/L | 56% | 80% |
| Salam (2018) | < 4.6 | U/L | 89% | 71% |
| **BALP** | | | | |
| Lima (2019) | < 27 | U/L (activity) | 79% | 70% |
| Sprague (2016) | < 33.1 | U/L (activity) | Not reported | Not reported |
| Jorgensen (2022) | < 24.2 | ug/L (mass) | 87% | 58% |
| Salam (2018) | < 21 | ug/L (mass) | 89% | 77% |
| Coen (1998) | < 12.9 | ug/L (mass) | 100% | 94% |
| **PINP** | | | | |
| Jorgensen (2022) | < 49.8 | ng/mL (intact) | 80% | 70% |
| Salam (2018) | < 57 | ng/mL (intact) | 80% | 75% |
| Salam (2018) | < 124 | ng/mL (total) | 80% | 68% |
| Sprague (2016) | < 498.9 | ng/mL (total) | Not reported | Not reported |
| **Combined** | | | | |
| Sprague (2016) | combined BALP + intact PTH | | Not reported | Not reported |
| Jorgensen (2022) | combined BALP + PINP (intact) | | 63% | 97% |
| Jorgensen (2022) | combined BALP + TRAP | | 63% | 91% |
| Jorgensen (2022) | combined PINP (intact) + TRAP | | 82% | 94% |

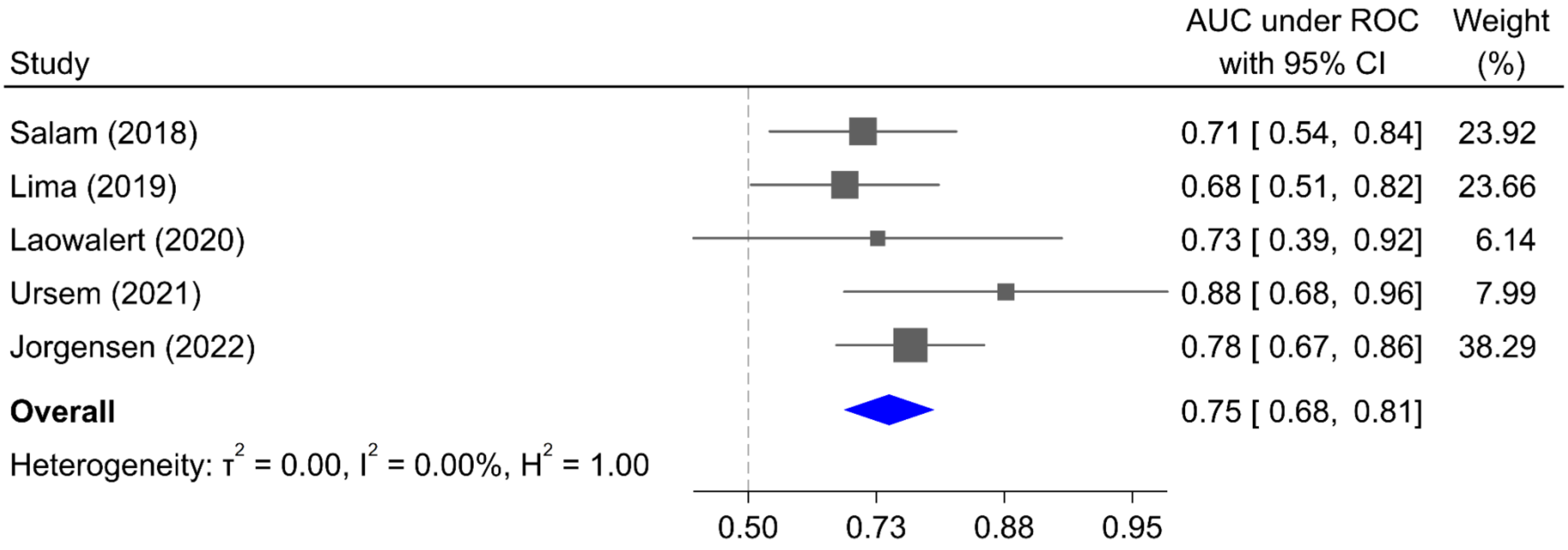


**Figure S1** Meta-analysis of TRAP5b for differentiating high vs non-high bone turnover status

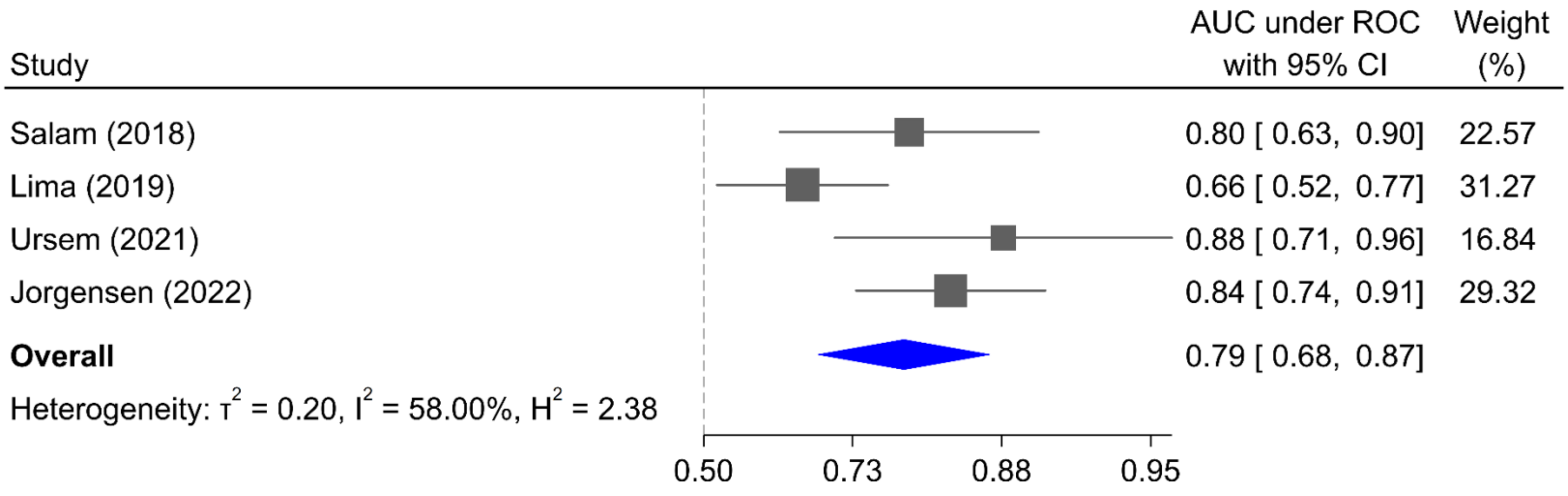


**Figure S2** Meta-analysis of TRAP5b for differentiating low vs non- low bone turnover status

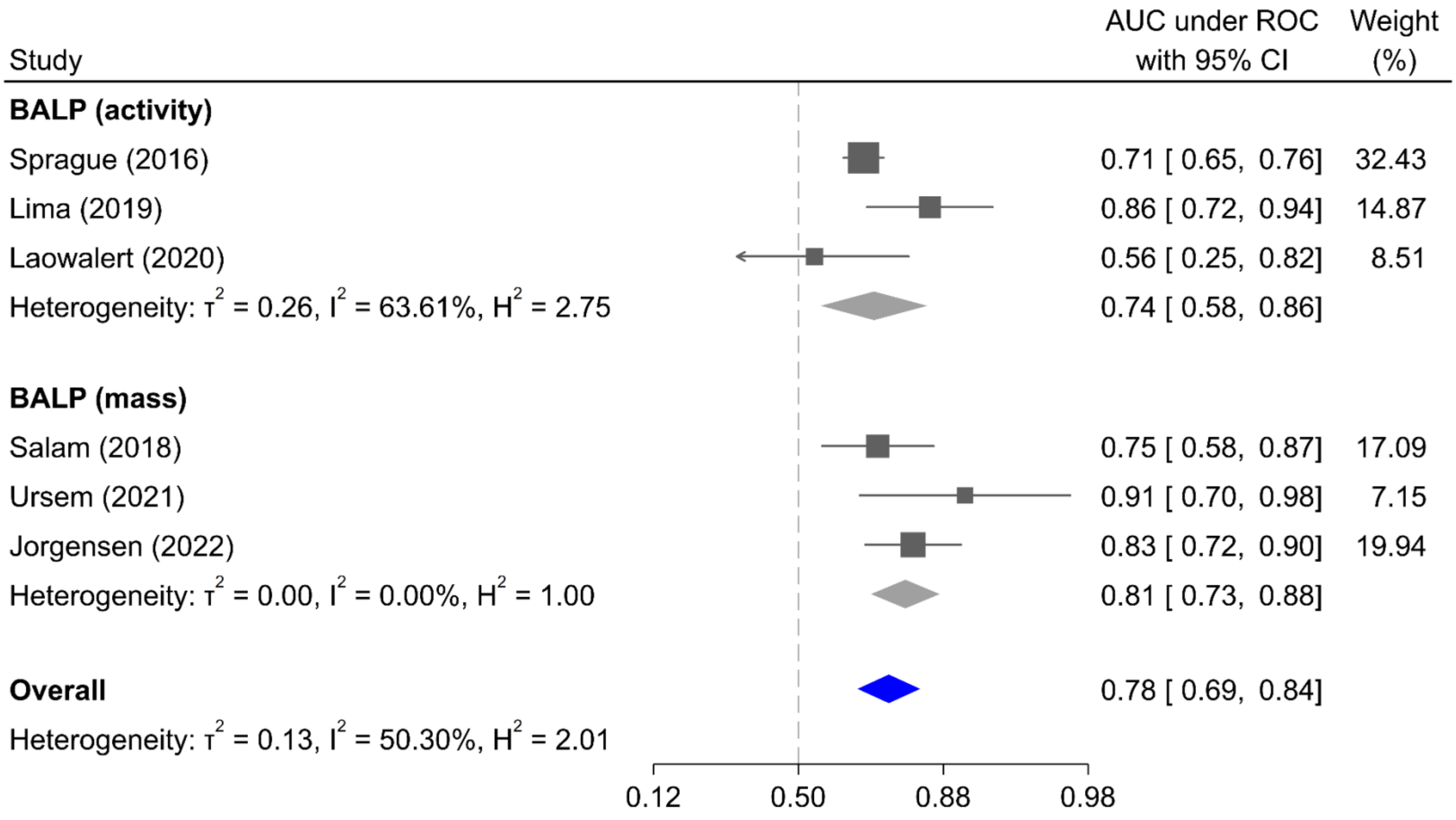


**Figure S3** Meta-analysis of BALP for differentiating high vs non-high bone turnover status

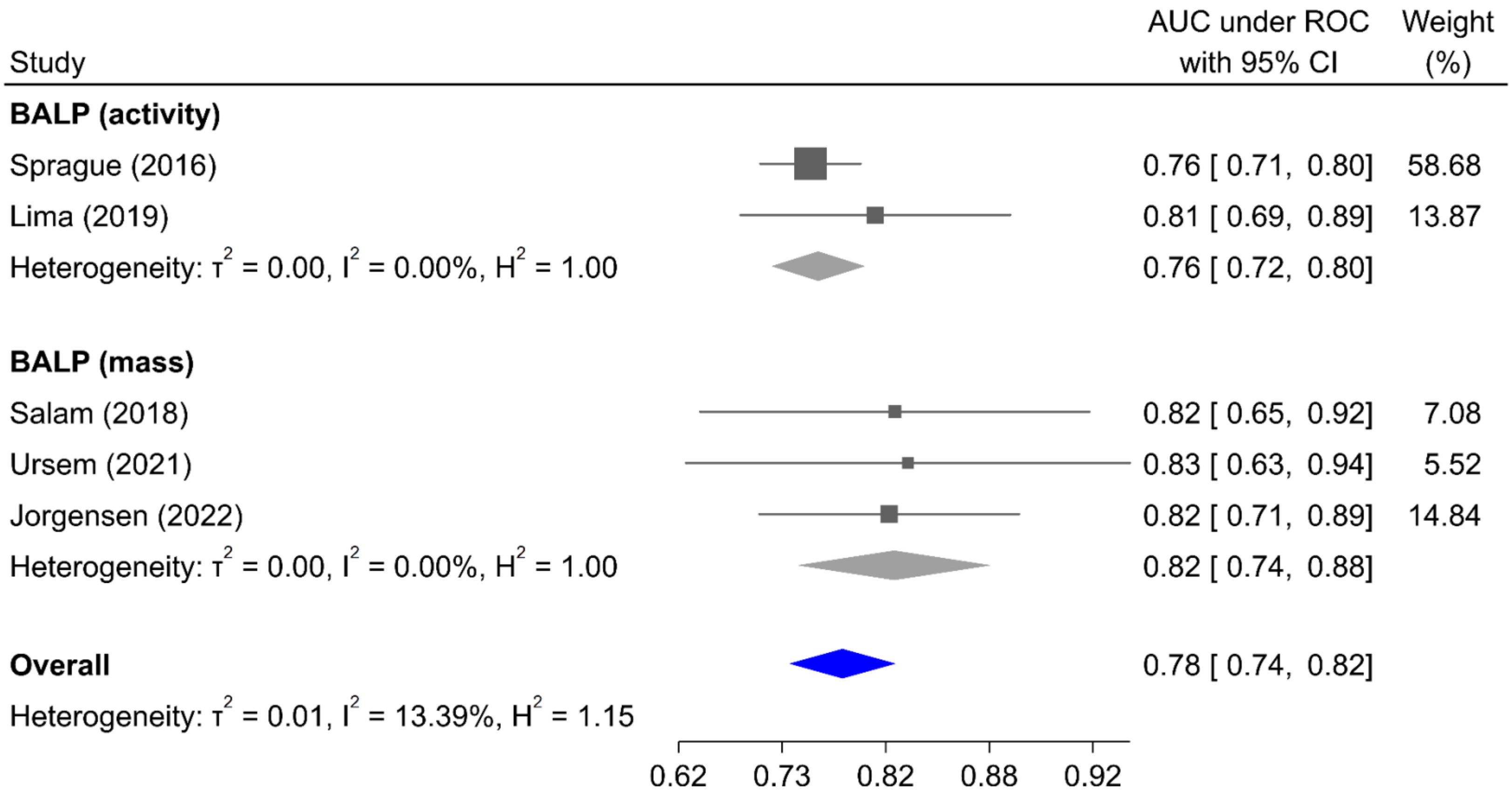


**Figure S4** Meta-analysis of BALP for differentiating low vs non- low bone turnover status

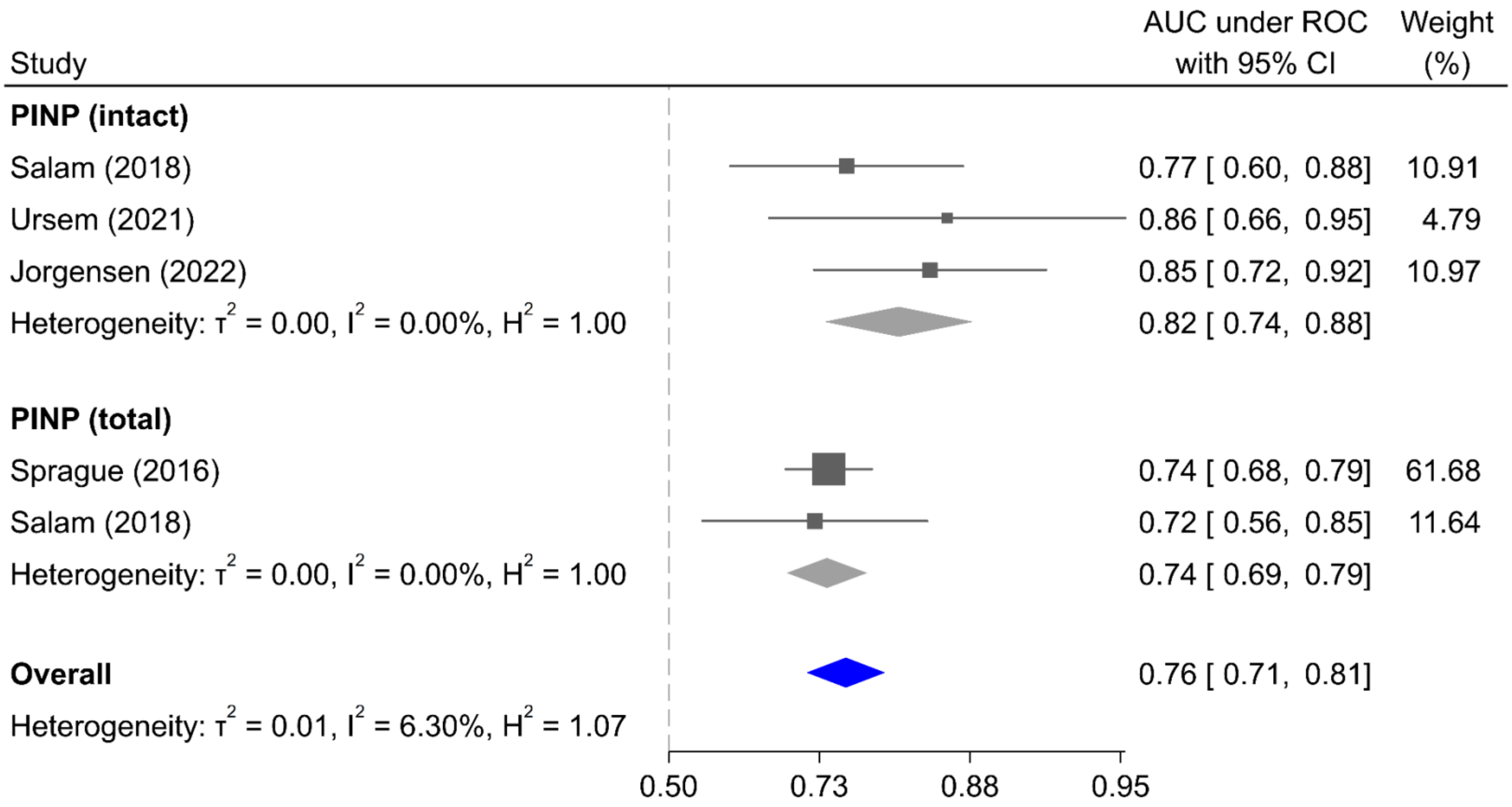


**Figure S5** Meta-analysis of PINP for differentiating high vs non-high bone turnover status

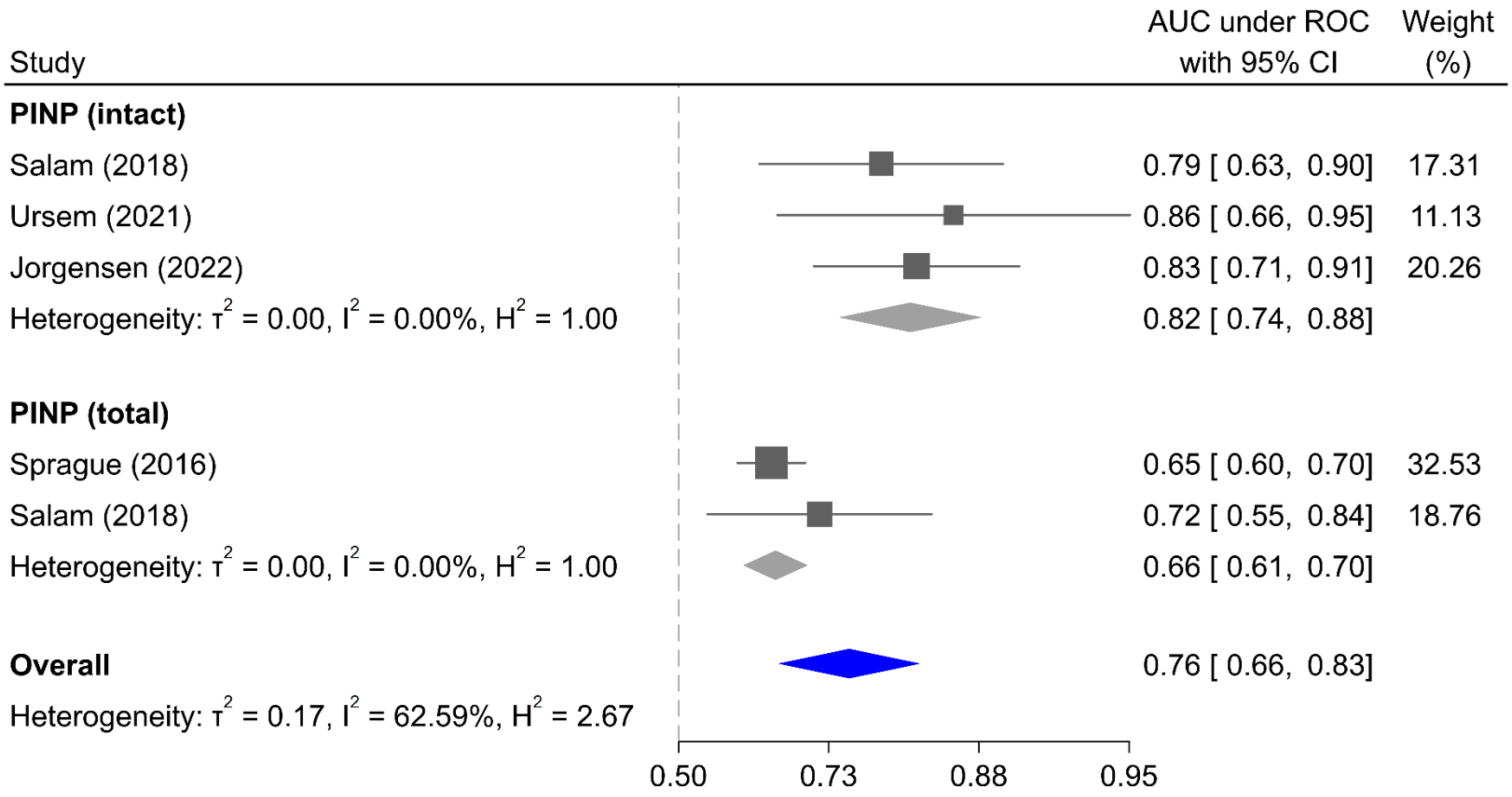


**Figure S6** Meta-analysis of PINP for differentiating low vs non- low bone turnover status

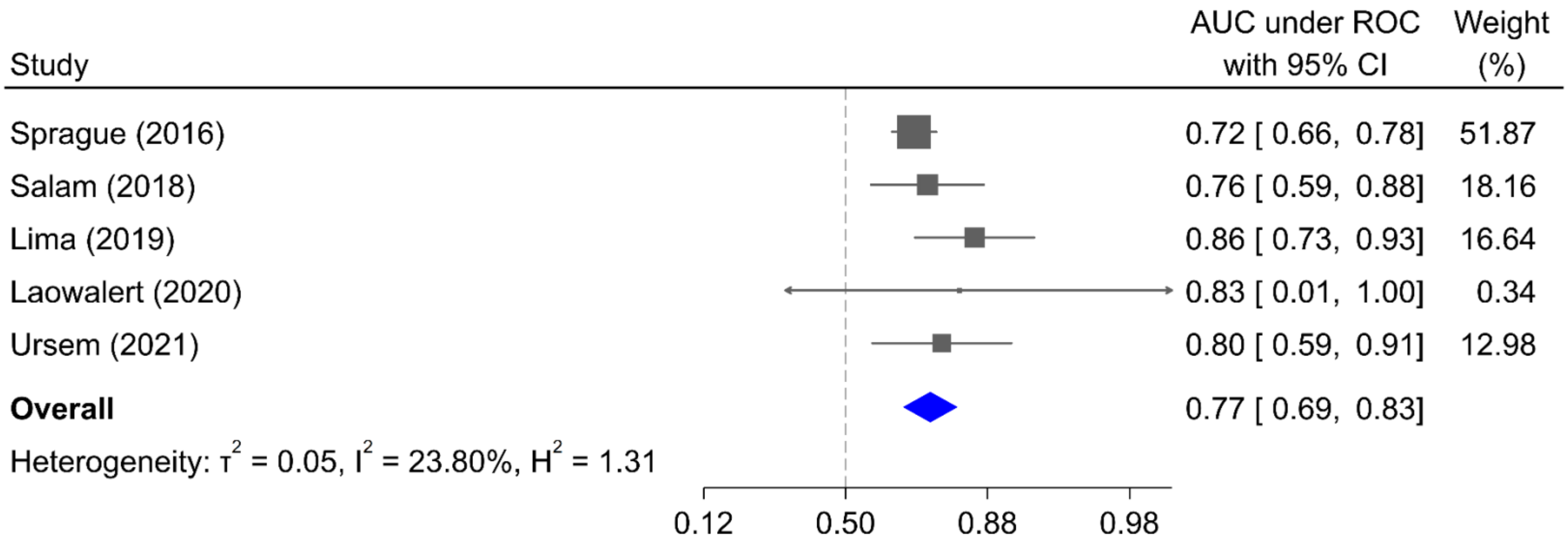


**Figure S7** Meta-analysis of intact PTH for differentiating high vs non-high bone turnover status

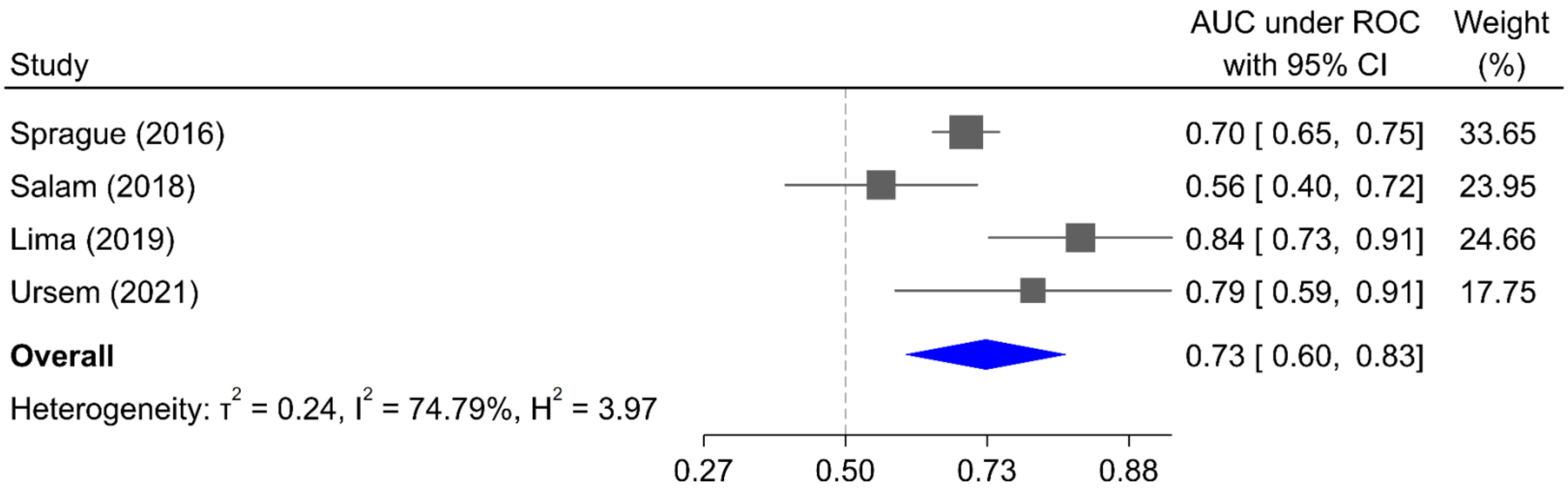


**Figure S8** Meta-analysis of intact PTH for differentiating low vs non- low bone turnover status
